# Effects of* Withania somnifera* on Reproductive System: A Systematic Review of the Available Evidence

**DOI:** 10.1155/2018/4076430

**Published:** 2018-01-24

**Authors:** Ramin Nasimi Doost Azgomi, Afshar Zomorrodi, Hossein Nazemyieh, Seyed Mohammad Bagher Fazljou, Homayoun Sadeghi Bazargani, Fatemeh Nejatbakhsh, Arezoo Moini Jazani, Yadollah Ahmadi AsrBadr

**Affiliations:** ^1^Department of Iranian Traditional Medicine, School of Traditional Medicine, Tabriz University of Medical Sciences, Tabriz, Iran; ^2^Department of Urology, Emam Reza Hospital, Tabriz University of Medical Sciences, Tabriz, Iran; ^3^Research Center for Pharmaceutical Nanotechnology, Faculty of Pharmacy, Tabriz University of Medical Sciences, Tabriz, Iran; ^4^Road Traffic Injury Research Center, Tabriz University of Medical Sciences, Tabriz, Iran; ^5^Department of Statistics and Epidemiology, Faculty of Health, Tabriz University of Medical Sciences, Tabriz, Iran; ^6^Department of Iranian Traditional Medicine, School of Traditional Medicine, Tehran University of Medical Sciences, Tehran, Iran; ^7^Department of Urology, Sina Hospital, Tabriz University of Medical Science, Tabriz, Iran

## Abstract

**Introduction:**

* Withania somnifera* (WS) also known as ashwagandha is a well-known medicinal plant used in traditional medicine in many countries for infertility treatment. The present study was aimed at systemically reviewing therapeutic effects of WS on the reproductive system.

**Methods:**

This systematic review study was designed in 2016. Required data were obtained from PubMed, Scopus, Google Scholar, Cochrane Library, Science Direct, Web of Knowledge, Web of Science, and manual search of articles, grey literature, reference checking, and expert contact.

**Results:**

WS was found to improve reproductive system function by many ways. WS extract decreased infertility among male subjects, due to the enhancement in semen quality which is proposed due to the enhanced enzymatic activity in seminal plasma and decreasing oxidative stress. Also, WS extract improved luteinizing hormone and follicular stimulating hormone balance leading to folliculogenesis and increased gonadal weight, although some animal studies had concluded that WS had reversible spermicidal and infertilizing effects in male subjects.

**Conclusion:**

WS was found to enhance spermatogenesis and sperm related indices in male and sexual behaviors in female. But, according to some available evidences for spermicidal features, further studies should focus on the extract preparation method and also dosage used in their study protocols.

## 1. Introduction

Infertility is a complicated problem with physiologic, psychologic, and economic aspects. Infertility is defined as the inability to conceive after one year of unprotected sexual intercourse [1]. About 15 percent of couples worldwide suffer infertility [2]. One in six couples is involved with infertility during their reproductive age. Based on World Health Organization report, 60–80 million couples suffer from failure of fertility worldwide [3]. It may be difficult to diagnose the reason for infertility but it may include either dysregulation of sex hormonal axis in both men and women or anatomical anomalies [4]. According to different studies, approximately 20%–50% of infertility is due to male, 40% is due to female factors, and 25% of causes are unknown [1, 5].

The causes of male infertility are classified as pretesticular, testicular, posttesticular, and unknown. Sperm abnormality causes 30%–40% of all infertility [6]. Pretesticular causes include disorders in the hypothalamus-pituitary-gonadal axis, systemic diseases, sexual dysfunction, and psychopathy. Testicular dysfunction due to multiple reasons as infection, trauma, varicocele, cryptorchidism, chromosomal anomalies, alcohol, cigarettes, drugs, and radiation is another cause of male infertility. Posttesticular disorders comprise the abnormalities in sperm transfer such as obstruction or dysfunction of epididymis and ductus deferens, immunological defects, and anatomical abnormalities like hypospadias [7, 8].

Different etiologies of female infertility include ovarian diseases, tubal disorders, endometriosis, uterine pathologies, cervical problems, congenital anomalies, and dysfunction of the hypothalamus-pituitary-ovarian axis and systemic diseases [1, 9]. Treatment of infertility may vary due to the different etiologies but it ranges from simple pharmacological treatments to advanced laboratory procedures and surgeries. In developing countries, due to the lack of adequate equipment to diagnosis and treatment for many infertility causes and also probable long-term diagnosis process, many people tend to use alternative and complementary medicine [10]. Herbal medicines are one of the main modalities used in this field.

*Withania somnifera*, (WS) also known as ashwagandha, Indian ginseng, winter cherry, horse smell, Kaknaje Hindi, is a well-known medicinal plant in Solanaceae family used in traditional medicine in many countries such as Iran and India [11]. This plant is known to cure impotency and increase sex appeal and fertility when used solitarily or in combination with other medications [12, 13]. This wild plant grows in dry and hot-semiarid climate such as southern Mediterranean region, Canary Islands, and northern Africa to northern India (Iran, Jordan, Sudan, Palestine, Afghanistan, and Egypt) [14, 15]. Different parts of this plant such as roots, leaves, flowers, seeds, stems, and fruits are used as remedy in traditional medicine of different countries [16–18]. Many phytochemicals have been extracted so far from this plant with possessing different pharmacologic and biological properties [19].

WS has been recommended for management of polyarthritis, lumbago, painful swellings, premature ejaculation, oligospermia, plague, asthma, vitiligo, general debility, impotency, ulcers, uterine infection, leucorrhoea, hemorrhoid, and orchitis in traditional Persian medicine [60, 61]. All these therapeutic uses suggest its anti-inflammatory, aphrodisiac, semenogogue, and deobstruent features [62–65]. As far as there are no wide-spectrum and specific studies or systematic reviews about therapeutic effects of WS, on male and female reproductive system, the present study was trying to systemically review therapeutic effects of WS on reproductive system and fertility disorders.

## 2. Methods

### 2.1. Study Design and Search Strategy

In this systematic review which was performed in 2016, required data were gathered using databases such as Google Scholar, PubMed, Scopus, Web of Science, and Cochrane Library. The keywords used in present study were “*Withania somnifera*” (also equivalent terms), “fertility”, “conceive”, “infertility”, “women”, “men”, “female”, “male”, “semen”, “sperm”, “spermicidal”, “Sertoli”, “prolactin”, “follicular stimulating hormone”, “luteinizing hormone”, “testosterone”, “libido”, “aphrodisiac”, “behavior”, “sexual”, “spermatogenesis”, “reproduction”, “semenogogue”, “impotency”, “spermatozoa”, “estrogen”, “pregnancy”, “gonadotropin releasing hormone”, “testis”, “leydig”, and “ovarian”. The time period between 1965 and 2017 was selected. Also, to increase the scope of the study, manual search in some of the valid journal databases was performed. All in vitro or in vivo studies about the effects of WS on reproductive system and fertility among human or animal subjects were included in the study. Review studies, case reports, letter to editors, and short communications were excluded from the study.

To search for unpublished articles (grey literature), European Association for Grey Literature Exploitation (EAGLE) and Health Care Management Information Consortium (HMIC) were searched.

### 2.2. Articles Evaluation

The selected papers extracted from the databases were assessed by two investigators using Consort 2010 checklist. Discrepancies between the two raters were referred to the third investigator. First, the titles of all articles were reviewed to screen for eligibility and those found to be irrelevant with the objectives of the study were excluded from the study. In the later stages, the abstracts and full-text articles were, respectively, examined to identify and exclude those that did not match the inclusion.

### 2.3. Data Extraction

One reviewer extracted the data from the included studies while a second author checked the results. Any disagreements were resolved by a discussion of reviewers. Data for the primary objective of the review was collected from the full text of each publication and included the trial name, year of publication, type of study, sample size, results, and other characteristics.

### 2.4. Statistical Analysis

Statistical analysis was performed by SPSS software package version 16.0 for windows (SPSS Inc., Chicago, USA) [66]. Quantitative data are presented as mean ± standard deviation (SD), while qualitative data are demonstrated as frequency and percent (%).

## 3. Results

**Figure 1:**
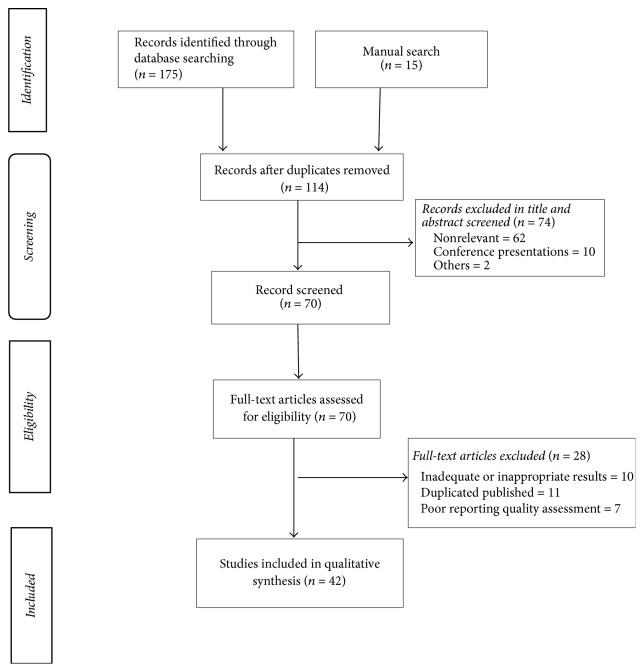
Flowchart of the systematic review process searching for studies investigating* Withania somnifera* on reproductive system.

The flowchart of the study is shown in Figure 1. Of 459 recognized studies, 42 studies were included in the present study. These studies were composed of 8 human studies (7 studies on men and one among women), 28 animal studies (20 studies of male animals and 8 studies on female animals), 5 animal-cellular studies, and one cellular study. In these studies, roots (29 studies), leaves (7 studies), fruits (2 studies), unknown extract (2 studies), and stems (1 study) were used. Characteristics and results of human, animal, and animal-cellular studies are shown in Tables 1, 2, and 3, respectively. In all human studies, root extract was used. The duration of human studies ranged from 60 to 90 days. Mostly the WS extract was used orally and once daily in 50 percent of human studies. In the most studies, no side effects were found for WS extract during of studies [17, 20, 21, 30, 36, 40, 45, 46, 58].

Many phytochemicals have been extracted from WS, which includes alkaloids, flavonoids, steroidal lactones, saponins, neurotransmitters, essential and nonessential fatty acids, ergostane, and gamma amino butyric acid; of all these components, alkaloids, and withanolids such as withaferin A, withanosides, sitoindosides, beta-sitosterol, and various amino acids like alanine have more prominent effect on fertility status [20, 23–25, 28–30, 34, 38, 51, 55].

In animal studies, WS is known to have gonadotropic function which increases gonadal weight by growthing follicles size in female and also increasing seminiferous tubular cell layers in male animals [27, 28, 31, 33, 35, 37, 41, 57–59]. WS is found to improve spermatogenic activity which is proposed to be due to supporting hypothalamic-hypophysial-gonadal hormonal axis and testosterone balance in testes [15, 31, 42]. WS is found to compensate LH and FSH decrease or increase in diabetic Wistar rats [27, 29, 31, 38, 47]. Also, WS increases testosterone [15, 29, 38, 39, 44, 47] and progesterone [29, 38] in male rats and decreases triglyceride and cholesterol in both male and female rats [28, 29, 35, 38, 43, 52].

In a study by Shukla et al. about effects of WS on men, WS root powder was used for 3 months and it was shown that sperm parameters such as count and motility in sperm analysis had improved due to decrease apoptosis and reactive oxidative stress among men with normospermia and oligospermia; also copper, zinc, iron, and gold ions of seminal plasma had increased after the treatment and subsequently semen quality increased. This increase in semen quality is proposed to be due to the increase in essential neurotransmitters, metallothionein which has antioxidative function, and metal ions as cofactors for essential enzymes [23].

In two clinical trials, the effects of 5 grams of WS root for 3 months on semen parameters of infertile men were investigated. Improvement in semen quality, increased vitamins E, C, and A, and increased fertility were reported which is proposed to be due to the high amount of alkaloids, ergostane steroids, and essential amino acids in WS which improved detoxification, decreased oxidative stress, and restorated testosterone secretion [12, 25].

In another human study, treatment by WS aqueous extract in married healthy women increased their sexual function index and diminished sexual distress index statistically significant [21]. In two studies using WS root extract for men with psychologic erectile dysfunction, there was no statistically significant difference between the intervention and control group considering sexual function indices [22, 26]. Also, WS root extract was found to decrease prolactin level after 3 months of administration among infertile men [12, 24, 25].

In a study by Bhattarai et al. about effects of WS root extract, it was found that GABA mimetic features of this extract led to an increased activity of gonadotropin releasing hormone secreting neurons [54]. On the other hand, WS root extract was found to decrease libido and sexual function which led to impotency and erectile dysfunction in animal studies [30]. Ethanolic WS fruit and stem extract were found to induce infertility in male rats due to the decrease in sperm motility, count, and degeneration of seminiferous tubules, although this extract did not have an effect on sperm morphology [28, 53, 56].

Prabu et al. in a study on male rats found that hydroalcoholic WS root extract was found to decrease white blood cell and lymphocyte counts in blood, but no considerable effect on reproductive indices [40]. Alcoholic WS root extract can decrease estrogen and cholesterol level in female Wistar mice and recover corpus luteum, graafian follicles, and germinal epithelium which has been damaged due to the chlorpyrifos exposure [35].

WS leaves and roots have been found to improve oxidative stress indices such as an increase in superoxide dismutase, catalase, glutathione, lactate dehydrogenase, alanine, glutamine, phenylalanine, and decrease in cortisol and fructose [12, 22–25, 34, 36, 47, 51, 58].

In a study investigating effects of 6.5 mg of WS root extract on* Nile tilapia*, it was found that this extract with androgenic effects decreased prolactin level and estrogen level by inhibiting aromatase activity and induced male phenotype formation; this phenomenon was proposed to be due to components such as tannin, saponins, terpenoids, steroids, and flavonoids [46].

## 4. Discussion

Traditional and complementary medicine have been more popular nowadays to cure health related conditions [67]. This proposes a new strong potential in traditional and complementary medicine to come up with new medical combinations with fewer side effects [68–70]. Traditional Persian medicine is one of the most well-known categories of traditional medicine using herbal medicine as one of the main therapeutic modalities [71].

*Withania somnifera* is one of the herbal medicines widely used for the treatment of infertility and sexual dysfunction. This plant has been known to contain more than 80 types of phytochemicals such as steroidal and nonsteroidal alkaloids, steroidal lactones and saponins like isopelletierine, anaferin, anahygrine, hygrine, cuscohygrine, tropine, pseudotropine, withananine, ashwagandha, withaferins, withananinine, pseudowithanine, somnine, somniferine, somniferinine, 3-tropyltigloate, withanine, withasomine, visamine, mesoanaferine, sitoindoside (7–10), hentriacontane, amino acids such as aspartic acid, glycine, tryptophan, proline, alanine, tyrosine, hydroxyproline valine, cystine, glutamic acid, and cysteine, calcium, phosphorus, iron, flavonoids, starch, reducing sugars, proteolytic enzyme “chamase,” glycosides, dulcitol, and volatile oil. Of all these components, withaferin A and sitoindosides had the key role in WS therapeutic effects [11, 13, 23, 33, 34, 60, 61, 72].

Based on the present study, it was shown that extracts of WS fruits, leaves, stems, and especially roots enhance sperm quality indices such as motility and count in men [12, 20, 24, 25] and also decrease the effects of chemical toxins on gonads in both men and women [13, 15, 29, 33–38, 41, 44, 45, 59]. WS can increase gonadal weight in both sexes, enhance folliculogenesis and spermatogenesis, and improve LH, FSH, and testosterone balances [15, 27, 31, 35, 36, 38, 42, 44, 45]. Sexual behavior indices such as female sexual function index and female sexual distress index improve statistically significant after WS extract administration [21].

The mechanism of WS effect on the reproductive system is not known entirely yet, but this mechanism is proposed to be linked to the antioxidative features and ability to improve the hormonal balance of LH, FSH, and testosterone and improve detoxification process. Also, the GABA mimetic feature of WS extract is thought to play the main role in inducing gonadotropin releasing hormone secretion and improving hormonal balance [23, 27, 31, 34–36, 42, 44, 47, 51]. In the male reproductive system, it is assumed that WS by providing metal ions facilitates enzyme activities, modifies oxidative stress, and prevents cell apoptosis [23]. The root extract of WS has been shown to induce alanine transaminase activity which increases alanine in seminal fluid leading to a less oxidative stress index and improved semen quality [24]. Normalizing lactate, phenylalanine, glutamine, citrate, and histidine in seminal fluid are another feature of WS extract which improves enzymatic processes in tricarboxylic acid cycle (TCA) and fatty acid metabolism [12, 59]. On the other hand, some animal studies have suggested that WS extract may cause reversible spermicidal and infertilizing effect in male and delayed puberty in both sexes; this might be due to the dose, preparation method, adjuvant components, and duration of use designated in mentioned studies [28, 30, 49, 53, 56].

## 5. Conclusion

Based on the results, it deems that* Withania somnifera* has a positive effect in the treatment of infertility both in male and female. Although some studies proposed that WS extract might have infertilizing and spermicidal effect. Due to the growing interest in using herbal medicine especially those which possess the antioxidative and reproductive system supporting properties, further studies are needed to be designed with higher population and more-structured methodology so a more precise and decisive conclusion can be made.

## Figures and Tables

**Table 1:** Characteristics and results of human studies investigating effects of *Withaniasomnifera* on reproductive system.

Number	Author/year	Design	Participant	Intervention protocol	Duration of study	Results	Place	Part/ compound
(1)	Mahdi et al., 2011 [12]	Controlled, prospective, before and after clinical trial	Normozoospermic infertile men (*N* = 60); 3 groups; heavy smoker (*n* = 20), under psychological stress (*n* = 20), unknown etiology (*n* = 20), control fertile men (*n* = 60)	5 g/d/PO/single dose with milk	3 months	No marked change in semen volume, ↓ liquefaction time (*p* < 0.01, *p* < 0.05), ↑ sperm concentration (*p* < 0.01, *p* < 0.05), ↑ sperm motility in cigarette smokers and psychological stress groups (*p* < 0.05), ↑ LH, ↓ FSH, ↑ T, ↓ PRl, ↓ cortisol, ↓ LPO in seminal plasma and ↑ SOD in 3 groups (*p* < 0.01), ↑ CAT in psychological stress groups (*p* < 0.01), ↑ glutathione, ↑ ascorbic acid in psychological stress and unknown etiology groups (*p* < 0.01), ↑ vitamin E, ↑ vitamin A in 3 groups (*p* < 0.05) after treatment compared to before treatment, ↑ 14% rate of success of pregnancy in the partners	India	Root powder

(2)	Ambiye et al., 2013 [20]	Double-blind, randomized, placebo-controlled clinical trial (a pilot study)	Oligospermic infertile male (*N* = 46); 2 groups: WS (*n* = 21) and placebo (*n* = 25)	675 mg: 1 capsule of 225 mg/thrice a day/PO	90 days	↑ 53% semen volume, ↑ 167% sperm count, ↑ 57% sperm motility (*p* < 0.0001), ↑ 17% serum T (*p* < 0.01), ↑ 34% LH (*p* < 0.02) after 90 days of therapy compared to the baseline values on Day 0 GASE and GATE in WS-treated compared to placebo were excellent (68.75%)	India	Full spectrum root extract

(3)	Dongre et al., 2015 [21]	Double-blind, randomized, placebo-controlled clinical trial (a pilot study)	Healthy married women (*N* = 50); WS (*n* = 25); placebo (*n* = 25)	Capsules of 300 mg/ twice daily/PO after food	8 weeks	↑ total score (FSFI) (*p* < 0.001), ↑ FSFI domain score for “arousal” (*p* < 0.001), “lubrication” (*p* < 0.001), “orgasm” (*p* = 0.004), “satisfaction” (*p* < 0.001), ↑ FSDS score (*p* < 0.001), ↑ number of successful sexual encounters (*p* < 0.001) after treatment compared to placebo PGART and PGATT in both groups were excellent, no toxic effect during 8 weeks in WS-treated group	India	High-concentration root water extract

(4)	Mamidi et al., 2014 [22]	Randomized, single-blind, placebo-controlled, parallel-group study	Men with ED (*N* = 86); 2 groups: WS (*n* = 41) and placebo (*n* = 45)	Four tablets 500 mg thrice a day (6 g)/PO after food	60 days	↑ 10.52% on EDSI, ↑ 4.18% on IMHQOL, ↑ 39.22 on QEQ in WS group, ↑ 11.20% on EDSI, ↑ 5.95% on IMHQOL, ↑ 45.74% on QEQ (*p* < 0.001) in control group. No marked difference between two groups on all the scales (*p* > 0.05). No significant effect in length, circumference, angle of erection of penis after treatment between both groups	India	Root powder

(5)	Shukla et al., 2011 [23]	Controlled, prospective, before and after clinical trial	Infertile men (*N* = 75); 3 groups: NZ (*n* = 25), OZ (*n* = 25), AZ (*n* = 25) and control, healthy fertile men (*n* = 75)	5 g/day/single dose with milk	3 months	↓ Sperms apoptosis in NZ and OZ men, ↓ ROS of spermatozoa in OZ and AZ infertile men (*p* < 0.05), ↑ metal ions concentration (Cu^2+^, Zn^2+^, Fe^2+^, Au^2+^) of seminal plasma in 3 groups (*p* < 0.01) after treatment comparison to before treatment	India	Root powder

(6)	Gupta et al., 2013 [24]	Controlled, prospective, before and after clinical trial	Infertile men (*N* = 180); 3 groups: NZ (*n* = 60), OZ (*n* = 60), AZ (*n* = 60) and control, healthy fertile men (*n* = 50)	5 g/day/PO/ single dose with milk	3 months	↑ sperm concentration, ↑ sperm motility, ↓ LPO, ↑ LH, ↑ T (*p* < 0.01), ↓ FSH, ↓ PRL, ↑ ALT, ↑ AST, ↑ LDH, ↑ IDH, ↑ alanine, ↑ glutamate, ↑ citrate, ↑ GPC, ↑ histidine, ↓ phenylalanine in seminal plasma, after treatment compared to before treatment in NZ, OZ, AZ groups (*p* < 0.05)	India	Root powder

(7)	Ahmad et al., 2010 [25]	Controlled, prospective, before and after clinical trial	Infertile men (*N* = 75); 3 groups:; NZ (*n* = 25), OZ (*n* = 25), AZ (*n* = 25) and control, healthy fertile men (*n* = 75)	5 g/day/PO with milk	3 months	↑ sperm concentration, ↑ sperm count (*p* < 0.01), ↑ sperm motility, ↓ LPO (*p* < 0.01), ↑ SOD, ↓ protein carbonyl groups in seminal plasma (*p* < 0.01), ↑ CAT (*p* < 0.01), ↑ glutathione (*p* < 0.01), ↑ vitamins A, E, and C (*p* < 0.01), ↑ seminal fructose (*p* < 0.05), ↑ T (*p* < 0.01), ↑ LH, ↓ FSH, ↓ PRL in all of the groups of infertile men after treatment compared to before treatment, ↑ semen volume in NZ and OZ groups after treatments compared to before treatments	India	Root powder

(8)	Mamidi and Thakar, 2011 [26]	Randomized, single-blind, placebo-controlled, parallel-group study	Men with ED (*N* = 86); 2 groups: WS (*n* = 41) and control (*n* = 45)	Four tablets 500 mg/PO/thrice a day after food	60 days	↑ 12.6% IIEF in WS-treated and ↑ 19.11% in placebo group (*p* < 0.001), no significant effect in the management of psychogenic erectile dysfunction and IIEF in WS-treated group compared to placebo group (*p* > 0.05)	India	Root powder

ND, not determined; WS, *Withania somnifera*; FSH, follicular stimulating hormone; LH, luteinizing hormone; ALP, alkaline phosphatase; Cd, cadmium; GSH, glutathione peroxidase; SOD, superoxide dismutase; CAT, catalase; MDA, malondialdehyde; STD, seminiferous tubules diameter; T, testosterone; PRL, prolactin; LPO, lipid peroxidation; GABAAg, amino butyric acid; TG, triglyceride; EDSI, erectile dysfunction severity index; FSFI, female sexual function index; FSDS, female sexual distress scale; IMHQOL, Internet mental health quality of life scale; QEQ, quality of erection questionnaire; NZ, normozoospermic; OZ, oligozoospermic; AZ, asthenozoospermic; IDH, isocitrate dehydrogenase; LDH, lactate dehydrogenase; ALT, alanine aminotransferase; AST, aspartate aminotransferase; GPC, glycerophosphocholine; IIEF, international index of erectile function; PGART, patient's global assessment of response to therapy; PGATT, patient's global assessment of tolerability to therapy; GASE, global assessment scale for efficacy; GATE, global assessment scale for tolerability; ↑, increase; ↓, decrease.

**Table 2:** Characteristics and results of animal studies investigating effects of *Withaniasomnifera* on reproductive system.

Number	Author/year	Plant extract	Participant	Intervention protocol	Duration of studies	Results	Place
(1)	AL-Qarawi et al., 2000 [27]	Lyophilized aqueous extract	Immature female Wistar rats (*N* = 60); 6 groups: 17-day-old rats; G1 (10), G2 (10), control (*n* = 10), 25-day-old rats G1 (*n* = 10), G2 (*n* = 10), control (*n* = 10) G1 = *C*. *coccineum*G2 = *W*. *somnifera*	47 mg/100 g body weight/stomach tube	6 days	In 25-day-old rats; ↑ FSH levels (*p* < 0.05), ↑ ovarian weight and deep folliculogenesis and proliferation of granulosa (*p* < 0.01) In 17-day-old animals; ↑ body weight (*p* < 0.01) no significant changes in the ovarian weight and folliculogenesis and FSH and LH	Kingdom of Saudi Arabia

(2)	Mali et al., 2008 [28]	50% ethanolic extract of fruits	Fertility proven, adult healthy male albino rats	50 mg/kg/PO/day	60 days	↓ sperm motility, ↓ density of testicular and cauda epididymal sperms, ↓ weight of testes and seminal vesicle, ↓ ascorbic acid, ↓ sialic acid, ↓ cholesterol, ↓ protein, ↓ fructose, ↓ acid phosphatases, ↑ degenerative changes in the seminiferous tubules and germinal epithelium, ↓ spermatogenic elements, in treated rats	India

(3)	Belal et al., 2012 [29]	Root power	Adult male albino rats (*N* = 40); alloxan induced diabetes; 4 groups: control (*n* = 10), nondiabetic WS-treated (*n* = 10), diabetic rats (*n* = 10), diabetic group treated with WS (*n* = 10)	Mixed with basal diet at ratio of 6.25%/po	4 weeks	No significant alteration estrogen and cholesterol in treated with WS both diabetic and nondiabetic rats compared to controls groups (*p* < 0.05), ↑ progesterone in both diabetic and nondiabetic treated with WS (*p* < 0.05), ↑ T (*p* < 0.05), ↑ LH (*p* < 0.001) in nondiabetic treated with WS groups compared to the control group, ↓ TG in diabetic treated with WS compared to diabetic rats (*p* < 0.05), ↓ FSH in WS-treated groups (*p* < 0.05), not considered hypoglycemic effect	Egypt

(4)	Ilayperuma et al., 2002 [30]	Methanolic extract of roots	Proven fertility, adult male Wistar rats (*N* = 40); 4 groups: control 1, control 2 (*n* = 20) and WS 1, WS 2 (*n* = 20)	3000 mg/kg/PO/day	7 days	Considerable weakness in libido, sexual performance, sexual vigour, and penile erectile dysfunction, no marked change in SGOT, SGPT, urea nitrogen, pH of the seminal vesicular fluid, wet weight of the organs and no deaths in treated period	Sri Lanka

(5)	Abdel-Magied et al., 2001 [31]	Lyophilized aqueous extracts of leaves	Immature male Wistar rats (*N* = 30); 3 groups: control (*n* = 10),* C*. *coccineum *(*n* = 10), WS (*n* = 10)	47 mg/100 g body weight/d/stomach tube	6 days	↑ LH, ↓ FSH, ↓ T, ↑ testicular weight, ↑ STD, ↑ number of seminiferous tubular cell layers (CL) and diameters, ↑ spermatogenesis in treated groups compared to control group	Saudi Arabia

(6)	Dhas et al., 2015 [32]	Ethanolic extracts	Female and male fish (*Etroplussuratensis*); 4 groups: control and WS with different ratio + *M. pruriens* + *Moringa oleifera*	120-200-300 mg/kg WS thrice in a day	ND	↑ Gonadosomatic Index (GSI), ↑ fecundity, ↑ striping response, ↑ percentage of fertilization, ↑ percentage of hatching, ↓ percentage of deformed, ↑ formed larvae, ↑ volume of milt, ↑ number of sperm cell, ↑ percentage of sperm motility, ↑ sperm survival time, ↑ percentage of active sperm after treatment diets especially the EXD3 contain 300 mg/kg (*p* < 0.05)	India

(7)	Shaikh et al., 2015 [33]	Glycowithanolides extract of fresh leaves	Adult Swiss albino male mice (*N* = 32); 4 groups: D-galactose treated (*n* = 8), control (*n* = 8), protective (*n* = 8), curative (*n* = 8)	20 mg/kg/injected subcutaneously	20 days	↑ epididymal sperm count (*p* < 0.01), ↑ weight of testes and epididymis and body (*p* < 0.01) and returned to normal histology of testes in protective and curative group compared to D-galactose treated group and no significant increase in weight of testes, epididymis and seminal vesicle in the curative compared to protective group	India

(8)	Kumar et al., 2015 [15]	Ethanolic root extract	Males Charles Foster rats (*N* = 30); arsenic induced testicular toxicity; 3 groups: control (*n* = 6), arsenic and WS-treated (*n* = 24)	100 mg/Kg/PO/day	30 days	↑ sperm count, ↑sperm motility, ↑ T, ↓ LH, ↓ LPO (*p* < 0.001), normalization the spermatogenetic stages, after treatment compared to before treatment	India

(9)	Walvekar et al., 2013 [34]	Glycowithanolides extract of fresh leaves	Adult Swiss albino male mice (*N* = 20); 4 groups: D-galactose (*n* = 5), control (*n* = 5), protective (*n* = 5), curative (*n* = 5)	20 mg/kg injected subcutaneously	20 days	↓ total and mitochondrial LPO, ↓ fluorescence product in testes, epididymis and seminal vesicle in protective and curative groups (*p* < 0.0001) compared to D-galactose	India

(10)	Kumar et al., 2015 [35]	Alcoholic root extract (5)%	Female Swiss albino mice; chlorpyrifos induced toxicity ovaries; 4 groups: control, chlorpyrifos, WS, *curcuma*	50 mg/kg/day	8 weeks	↓ estrogen, ↓ cholesterol and restoration in germinal epithelium, graafian follicles and corpus luteum of ovary in WS-treated group compared to chlorpyrifos group	India

(11)	Patil et al., 2012 [36]	Ethanolic extract of fresh leaves	Healthy male albino mice (*N* = 12); D-galactose induced stress; 3 groups: D-galactose (*n* = 6), control (*n* = 6), WS + D-galactose (*n* = 6)	2% extract	15 days	↓ total and mitochondrial LPO, ↑ sperm count, recovery of degenerative changes in histological structure both testis and epididymis after the treatment WS compared with D-galactose group	India

(12)	Rajashree et al., 2011 [37]	Alcoholic root extract	Male albino rats (*N* = 24); streptozotocin-induced diabetic; 4 groups: STZ (*n* = 6), normal Control (*n* = 6), WS + STZ (*n* = 6), STZ + insulin (*n* = 6)	500 mg/kg/PO/day	30 days	↑ weight of testes, ↑ caudal sperm count, ↑ weight of cauda epididymis in WS-treated diabetics compared to insulin treated groups	India

(13)	Kiasalari et al. 2009 [38]	Root powder	Wistar male rats (*N* = 39): ZTZ induced diabetic; 4 groups: ZTZ (*n* = 9), control (*n* = 8), WS + STZ (*n* = 11), sham (*n* = 11)	Plant-mixed pelleted food at ratio of 6.25%/po/day	4 weeks	↓ FSH (*p* < 0.05), ↑ LH (*p* < 0.05), ↑ progesterone (*p* < 0.05), ↑ T (*p* < 0.05), nonsignificant changes on estrogen in somnifera-treated diabetic, nondiabetic group compared to nontreated diabetic and nondiabetic, ns difference glucose, cholesterol after WS treatment, ↓ TG in somnifera-treated diabetic group	Iran

(14)	Rahmati et al., 2016 [39]	Root powder	Male rats (*N* = 48); 4 groups: morphine induced addiction: addicted (*n* = 12), control (*n* = 12), WS-treated control (*n* = 12), WS-treated addicted (*N* = 12)	Plant-mixed pelleted food at ratio of 6.25% (0.3 ± 0.01 g/kg/day)	21 days	↑ estrogen in WS-treated control groups (*p* < 0.05) compared to control group, no marked effect in addict group, ↑ T, ↑ LH in WS-treated addicted group (*p* < 0.05) compared to control and addicted groups, ↑ FSH in WS-treated control group compared to control group (*p* < 0.05)	Iran

(15)	Prabu et al., 2014 [40]	Hydroalcoholic root extract	Male Wistar rats (*N* = 12); 2 groups: control (*n* = 6) and WS (*n* = 6)	1000 mg/kg/PO (gavage)	70 days	↓ WBC, ↓ LYM values (*p* < 0.05), ↑ insignificant neutrophil, ↑ RDW, ↓ monocyte, ↓ eosinophil, ↓ RBC counts, ↓ PCT values, no significant difference in body weight, testes and seminal vesicles weight, sperm count, morphology and biochemical parameters between treated group and control group (*p* < 0.05). No side effects in during treatment	India

(16)	Bhargavan et al., 2015 [41]	Ethanolic root extract	Healthy adult male Wistar rats (*N* = 24); 4 groups: ethanol induced oxidative damage in the testes (*n* = 6), control (*n* = 6), WS + alcohol (*n* = 6), WS (*n* = 6)	200 mg/PO/day	28 days	↑ testicular weight, ↑ body weight and recovered histopathological changes in the seminiferous tubules, ↑ sperm count, ↑ motility, ↓ sperm abnormality, ↓ MDA (*p* < 0.01), ↑ GSH (*p* < 0.01), ↑ CAT (*p* < 0.01), ↑ T in alcohol + WS cotreatment group compared with alcohol group	India

(17)	Nirupama and Yajurvedi, 2015 [42]	Chloroform and ethanolic extracts of roots	Adult male rats (*N* = 30); 6 groups: chronic stress exposed on testis (*n* = 5), control (*n* = 5), positive control (*n* = 5), stress + ethanolic WS (*n* = 5), stress + mifepristone (*n* = 5), stress + chloroform extract of WS (*n* = 5)	10 mg/kg/day/orally (intubation)	1 month	↑ testicular 3*β* HSDH activity, ↓ adrenal 3*β* HSDH activity (*p* < 0.05), ↓ abnormal sperm count (*p* < 0.05), ↓ MDA, ↑ T (*p* < 0.05), ↑ total epididymal sperm count (*p* < 0.05), ↑ spermatogenesis, ↑ (SOD, CAT, GPx, GST, GR), ↑ ascorbic acid, tocopherol in WS-treated group compared to stressed rats	India

(18)	Kumar et al., 2013 [43]	Alcoholic extract of root	Female Swiss albino mice (*N* = 36); chlorpyrifos induced toxicity of ovary; WS + chlorpyrifos (*n* = 30); control (*n* = 6)	50 mg/kg/day	8 weeks	↓ estrogen and cholesterol (*p* < 0.0001), recovery of ova, granulosa cells, germinal epithelium, mature graafian follicle, mitochondrial cristae of ovary and chromatin material after WS administration compared to before treatment	India

(19)	Kumar et al., 2012 [44]	Aqueous extract of roots	Male mice (*N* = 20); endosulfan exposed spermatozoa; 2 groups: control (*n* = 10) and endosulfan + WS (*n* = 10)	1000 mg/kg/day	8 weeks	↓ MDA, ↑ T, ↓ calcium, restoration of spermatozoa structure such as normal plasma and nuclear membrane, Golgi complex, chromatin material and mitochondrial cristae after WS treatment compared to before treatment	India

(20)	Kaspate et al., 2015 [45]	Hydroalcoholic extract of roots	Healthy female Wistar rats with tubal ligated (*N* = 24): 4 groups with various doses of WS	100, 200 and 300 mg/kg/PO/day	21 days	↑ sexual behavior, ↓ run time, ↑ proximity time, ↓ retreats (*p* < 0.05, *p* < 0.01), ↑ serum estradiol with doses 200 and 300 mg/kg (*p* < 0.05, *p* < 0.01), normalization in histology of genital organs like vagina, uterine horn and ovary in estrous female rats compared to estrous control female rats	India

(21)	De and Chakraborty, 2016 [46]	Root powder	Mixed-sex juveniles of *Niletilapia (Oreochromisniloticus)* (*N* = 360); 9 groups: control (*n* = 40) and WS with various concentration (*n* = 320)	0.0, 2.0, 3.0, 3.5, 5.0, 6.5, 7.0, 8.0, 9.5 g/kg mixed food	30 days	Concentrations up to 7.0 g/kg have no side effect (*p* < 0.05), ↑ percentage of male and ↓ percentage of female with any concentration of WS (*p* < 0.05), high percentage of survival and male were at the concentration of 6.5 g/kg compared to control group	India

(22)	Jasuja et al., 2013 [47]	Methanolic extract of leaves and roots	Male albino rats (*N* = 36); 6 groups: control (*n* = 6), acephate for 15 days (*n* = 6), acephate for 30 days (*n* = 6), acephate for 15 days + WS for 15 days (*n* = 6), acephate + WS for 30 days (*n* = 6), acephate for 15 days and no treatment for next 15 days (*n* = 6)	100 mg/kg/PO/day	15–30 days	↓ testicular LPO, ↑ GSH, ↑ SOD, ↑ CAT, ↑ T, ↑ FSH, ↑ LH (*p* < 0.01, *p* < 0.05), recovery of sperm count, motility, morphology, testis histology after WS treatment compared with acephate groups	India

(23)	Saritha et al., 2011 [48]	Leaf powder-mixed pelleted food	Female rats (*N* = 35); 5 groups: control (*n* = 7), 0.05% Pb + normal pellet diet (*n* = 7), 0.05% Pb + WS diet (*n* = 7), 0.15% Pb + normal pellet diet (*n* = 7), 0.15% Pb + WS diet (*n* = 7)	500 mg/kg/PO/day	45 days	↓ length of the diestrus phase (*p* < 0.001), ↑ the number of implantations (*p* < 0.001), ↑ the number of live foetuses (*p* < 0.001), ↓ pre- and postimplantation losses after treatment with WS compared with the rats exposed and no toxicity in all of animals during treatment	India

(24)	Garg and Parasar, 1965 [49]	Root power	Albino mice of either sex (*N* = 16); 2 groups: control (*n* = 8) and WS group (*n* = 8)	25 mg/PO/day	10 days	↓ fertility rate by 25%, ↓ mating behavior, ↓ number of pups per litter from 5.25 to 3.0 in WS treatment group compared to control group	India

(25)	Sahin et al., 2016 [50]	Hydroalcoholic extract of roots	Male rats (*N* = 35); 5 groups: control (*n* = 7), sildenafil-treated (*n* = 7), *Mucuna* (*n* = 7), *Tribulus* (*n* = 7), WS (*n* = 7)	300 mg/kg/PO/day	8 weeks	No significant changes in body weight and reproductive organ weights, abnormal sperms, serum biochemical and hematology parameters (*p* > 0.05), ↓ mounting latency and intromission latency (*p* < 0.0001), ↑ mounting frequency and intromission frequency values (*p* < 0.0001), ↑ sperm counts, ↑ sperm motility, ↓ MDA, ↑ T (*p* < 0.0001) in WS groups compared to control group	Turkey

(26)	Shaikh et al., 2014 [51]	Glycowithanolides extract of fresh leaves	Adult Swiss albino male mice (*N* = 32); 4 groups: D-galactose treated (*n* = 8), control (*n* = 8), protective (*n* = 8), curative (*n* = 8)	20 mg/kg/injected subcutaneously	20 days	↑ SOD, ↑ GPx, ↑ CAT in testes and accessory reproductive organs (*p* < 0.01) in protective and curative group compare to D-galactose group	India

(27)	Saiyed et al., 2016 [52]	Hydroalcoholic extract of roots	Female Wistar rats (*N* = 24); letrozole induced PCOS: 4 groups: negative control (*n* = 6), positive control (*n* = 6), WS and TT (*n* = 6), standard group with clomiphene citrate (*n* = 6)	198 mg/kg	28 days	↑ number of days in estrus phase (*p* < 0.05), ↓ duration of diestrus phase in test group (*p* < 0.01) returned to normalcy on ↑ FSH, ↓ LH, ↓ T, ↓ estradiol and recovery of ovary and uterine weight but no significant, ↓ cholesterol (*p* < 0.01) compared to positive control group	India

(28)	Mali, 2013 [53]	Hydroalcoholic extract of fruits	Healthy, male albino rats (*N* = 12); 2 groups: control (*n* = 6) and WS (*n* = 6)	200 mg/kg/day/po	60 days	↓ primary and secondary spermatocytes, mature sperms, ↓ weight of testis and other accessory reproductive organs, ↑ abnormal seminiferous tubules, ↓ protein, ↓ sialic acid, ↓ fructose, ↓ ascorbic acid (*p* < 0.01, *p* < 0.001) in WS groups compared to control group	India

ND, not determined; WS, *Withania somnifera*; FSH, follicular stimulating hormone; LH, luteinizing hormone; ALP, alkaline phosphatase; Cd, cadmium; GSH, glutathione peroxidase; SOD, superoxide dismutase; CAT, catalase; MDA, malondialdehyde; STD, seminiferous tubules diameter; T, testosterone; PRL, prolactin; LPO, lipid peroxidation; GABA_A_ g, amino butyric acid; TG, triglyceride; EDSI, erectile dysfunction severity index; FSFI, female sexual function index; FSDS, female sexual distress scale; IMHQOL, Internet mental health quality of life scale; QEQ, quality of erection questionnaire; NZ, normozoospermic; OZ, oligozoospermic; AZ, asthenozoospermic; IDH, isocitrate dehydrogenase; LDH, lactate dehydrogenase; ALT, alanine aminotransferase; AST, aspartate aminotransferase; GPC, glycerophosphocholine; TT, *Tribulus terrestris; *WBC, white blood cell*; *LYM,lymphocytes*.; *↑, increase*; *↓, decrease.

**Table 3:** Characteristics and results of animal-plant and cellular studies investigating effects of *Withaniasomnifera* on reproductive system.

Number	Author/year	Type of study	Participant	Intervention protocol	Plant/extract	Duration of studies	Results	Place
(1)	Bhattarai et al., 2010 [54]	In vitro	GnRH neurons of male and female juvenile mice brain (*N* = 20) under patch clamp technique	Bath application of the 400 ng/*μ*l under condition of high cl^−^	Methanolic extract of root powder	ND	Production of potent membrane depolarization of the GnRH neurons, ↑ spontaneous action potentials, ↑ GABA_A_ memetic activity	Republic of Korea

(2)	Kataria et al., 2015 [55]	In vitro & in vivo (animal)	Rat hypothalamic GnV-3 cells & Wistar adult male rats (*N* = 6); 2 groups: control (*n* = 3) and WS (*n* = 3)	In vitro: 0.05–1.5% ASH-WEX In vivo: 4 ml/kg ASH-WEX/oral route	Aqueous extract of leaves	24 h & 21 days	In vitro: significant changes in morphology and physiological in GnV-3 as cell body size and neurite process, ↓ LDH levels at higher concentrations of WS, ↑ release of GnRH extra cellularly in the GnV-3 cells after treatment with WS, no elevated cytotoxicity, viable of 61.2% of cell after WS In vivo: no marked difference in GnRH level in WS-treated as compared to control group but upregulation of GnRH expression after treatment with WS	India

(3)	Singh et al., 2013 [56]	In vitro & in vivo (animal)	Rat semen & proven fertility, male albino rat (*N* = 18); 3 groups: control (*n* = 6), WS 25 mg (*n* = 6), WS 50 mg (*n* = 6)	2, 4, 6, 8, 10, 25 & 50 mg/kg/day/orally	Ethanolic extract of stems	20 s & 60 days	In vitro: minimum effective concentration of WS to kill 1 million sperm in 20 s was around 10 ± 0.066 In vivo: ↓ sperm density of cauda epididymal sperms, ↓ weight of testes, ↓ epididymis and seminal vesicle, ↓ spermatogenesis, ↓ sperm motility of cauda epididymal sperms, ↓ seminiferous tubules size, ↓ leydig cell nuclei diameter, ↓ seminiferous tubular diameter, ↓ rate of fertility in high dose WS and no considerable changes in T, FSH, sperms morphology, serum biochemistry, hematological parameters, body weight compared to control group	India

(4)	Ganu et al., 2010 [57]	In vitro & in vivo (animal)	Healthy male rats (*N* = 6) & adult male mice (*N* = 48); 8 groups: control (*n* = 6), abutilon indicum with various doses (*n* = 18), WS with various doses (*n* = 18), sildenafil (*n* = 6)	1 mg/ml & 100, 200, 400 mg/kg/PO	Aqueous extract of roots	28 d	↑ sperm count in all groups (*p* < 0.01), ↑ mounting frequency (*p* < 0.05) with WS 200, 400, ↑ frequency of penile erection episodes (*p* < 0.05), ↑ number of female licking behaviors with WS 400 (*p* < 0.01), ↑ mating behavior with WS 400 (*p* < 0.05), ↑ body weight with WS 400 (*p* < 0.01), ↑ testes weight, ↑ weight of prostate with WS 400, 200 (*p* < 0.01) compared to control	India

(5)	Prithiviraj et al., 2013 [58]	In vitro & in vivo (animal)	Male Wistar albino rats (*N* = 30); 5 groups: cadmium-induced oxidative injury in testis (*n* = 6), control (*n* = 6), cadmium + WS (*n* = 6), cadmium and vitamin E (*n* = 6), control and WS (*n* = 6)	300 mg/kg dissolved in 2% gum acacia /gavage	Root power	30 days	↑ SOD (*p* < 0.05), ↑ CAT (*p* < 0.01), ↑ GPX (*p* < 0.01), ↑ GSH (*p* < 0.001), ↑ levels of Vit C (ascorbic acid) (*p* < 0.05), Vit E (*α*tocopherol) (*p* < 0.01), ↓ ROS, ↓ LPO, ↓ caspase-3 in Cd + WS testis compared to Cd testis, ↓ GST (*p* < 0.05) in Cd + WS compared to normal level, ↓ apoptotic cells (*p* < 0.001), ↑ weight and volume of testes, ↑ leydig cell number (*p* < 0.05), ↓ necrotic or pathological change of testes in Cd + WS compared to control, no toxic side effect, stress, changes in behavior	India

(6)	Kyathanahalli et al., 2014 [59]	In vitro & in vivo (animal)	Prepubertal male rats (*N* = 18); 3 groups: streptozotocin-induced testicular oxidative impairments (*n* = 6), control (*n* = 6), STZ + WS (*n* = 6)	500 *μ*g & 500 mg/kg/day/oral gavage	Aqueous root extract	15 days	In vitro: considerable inhibition of deoxyribose and stable free radical DPPH In vivo: ↓ LPO, ↓ ROS in testis cytosol 38%, mitochondria 24% of STZ + WS groups but no marked change, ↑ total Thiol (TSH, GSH), ↑ nonprotein thiol (NPSH) in testis cytosol and mitochondria, ↑ weights of testis, ↓ blood glucose of STZ + WS group compared to STZ group (*p* < 0.05)	India

ND, not determined; WS, *Withania somnifera*; FSH, follicular stimulating hormone; LH, luteinizing hormone; ALP, alkaline phosphatase; Cd, cadmium; GSH, glutathione peroxidase; SOD, superoxide dismutase; CAT, catalase; MDA, malondialdehyde; STD, seminiferous tubules diameter; T, testosterone; PRL, prolactin; LPO, lipid peroxidation; GABAA g-amino butyric acid; TG, triglyceride; EDSI, erectile dysfunction severity index; FSFI, female sexual function index; FSDS, female sexual distress scale; IMHQOL, Internet mental health quality of life scale; QEQ, quality of erection questionnaire; NZ, normozoospermic; OZ, oligozoospermic; AZ, asthenozoospermic; IDH, isocitrate dehydrogenase; LDH, lactate dehydrogenase; ALT, alanine aminotransferase; AST, aspartate aminotransferase; GPC, glycerophosphocholine; ↑, increase; ↓, decrease.
