# Corrigendum to “Effects of *Withania somnifera* on Reproductive System: A Systematic Review of the Available Evidence”

**DOI:** 10.1155/2019/7591541

**Published:** 2019-11-21

**Authors:** Ramin Nasimi Doost Azgomi, Afshar Zomorrodi, Hossein Nazemyieh, Seyed Mohammad Bagher Fazljou, Homayoun Sadeghi Bazargani, Fatemeh Nejatbakhsh, Arezoo Moini Jazani, Yadollah Ahmadi AsrBadr

**Affiliations:** ^1^Department of Iranian Traditional Medicine, School of Traditional Medicine, Tabriz University of Medical Sciences, Tabriz, Iran; ^2^Department of Urology, Emam Reza Hospital, Tabriz University of Medical Sciences, Tabriz, Iran; ^3^Research Center for Pharmaceutical Nanotechnology, Faculty of Pharmacy, Tabriz University of Medical Sciences, Tabriz, Iran; ^4^Road Traffic Injury Research Center, Tabriz University of Medical Sciences, Tabriz, Iran; ^5^Department of Statistics and Epidemiology, Faculty of Health, Tabriz University of Medical Sciences, Tabriz, Iran; ^6^Department of Iranian Traditional Medicine, School of Traditional Medicine, Tehran University of Medical Sciences, Tehran, Iran; ^7^Department of Urology, Sina Hospital, Tabriz University of Medical Science, Tabriz, Iran

In the article titled “Effects of *Withania somnifera* on Reproductive System: A Systematic Review of the Available Evidence [1],” there were errors in the Results section and Figure 1, which should be corrected as follows:

In the Results section, the sentence “Of 459 recognized studies, 42 studies were included in the present study” should be “Of 190 recognized studies, 42 studies were included in the present study”.

In Figure 1, “Records after duplicates removed (*n* = 114)” should be “Records after duplicates removed (*n* = 144).” The corrected Figure 1 is as follows:

## Figures and Tables

**Figure 1:**
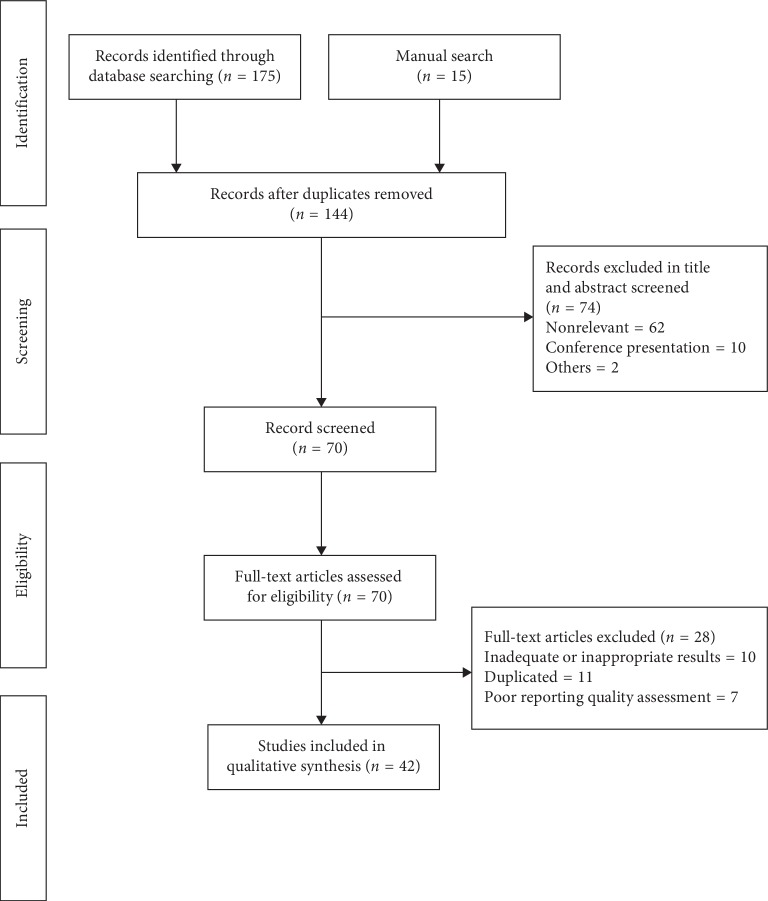
Flowchart of the systematic review process searching for studies investigating *Withania somnifera* on the reproductive system.
